# Prognostic and predictive role of tumour-associated macrophages in HER2 positive breast cancer

**DOI:** 10.1038/s41598-019-47375-2

**Published:** 2019-07-29

**Authors:** Tiia J. Honkanen, Antti Tikkanen, Peeter Karihtala, Markus Mäkinen, Juha P. Väyrynen, Jussi P. Koivunen

**Affiliations:** 1 Department of Oncology and Radiotherapy, Oulu University Hospital, POB 20, 90029 Oulu, Finland; 2 Department of Pathology, Oulu University Hospital, POB 21, 90029 Oulu, Finland; 3 Medical Research Center Oulu, POB 5000, 90014 Oulu, Finland; 4 Cancer and Translational Medicine Research Unit, University of Oulu, POB 5000, 90014 Oulu, Finland; 5 Department of Oncologic Pathology, Dana-Farber Cancer Institute and Harvard Medical School, 450 Brookline Ave, Boston, MA 02215 USA

**Keywords:** Tumour biomarkers, Breast cancer, Tumour immunology, Targeted therapies, Breast cancer

## Abstract

Disease outcomes of HER2+ breast cancers have dramatically improved after targeted therapies, such as trastuzumab became available. The main mechanism of action of trastuzumab depends on immunoactivation, while immunosuppressive tumour phenotype has been linked to adverse outcomes. Current study included metastatic HER2+ breast cancer patients treated with trastuzumab (n = 40). Immunohistochemistry was conducted to detect nitric oxide synthase 2 (iNOS) expressing M1 polarized and CD163^+^ M2 polarized macrophages, FoxP3^+^ regulatory T-cells (Tregs), CD47 and indoleamine 2,3-dioxygenase 1 (IDO1). High number of iNOS^+^ M1-like macrophages, both in the center of the tumour (CT) and invasive margin (IM), was significantly associated with improved survival (p = 0.009) while high expression of IDO1 or CD47 in the malignant cells was associated with worsened prognosis (p = 0.018, p = 0.046). High number of CD163^+^ M2-like macrophages in the CT, but not in the IM, and high number of FoxP3^+^ Tregs in both locations showed non-significant tendencies towards poor prognosis. Moreover, high number of iNOS^+^ M1-like macrophages combined with high number of CD8^+^ T-cells in the CT was significantly associated with improved survival (p = 0.0003), and this combined marker predicted patient’s ability to remain progression-free without trastuzumab after responding to the therapy (p = 0.003). Current study highlights the role of M1 polarized macrophages alone and in combination with CD8^+^ cells in HER2+ breast cancer.

## Introduction

Human epidermal growth factor receptor 2 (*HER2*) gene is frequently amplified in breast cancers. Before introduction of modern HER2 targeted therapies such as trastuzumab, a monoclonal HER2 antibody, *HER2* amplified disease was associated with high risk for relapse in localized disease and shortened overall survival in metastatic disease^1–5^. Trastuzumab functions by binding to the HER2 receptor and its main effect is mediated through antibody-dependent cellular cytotoxicity and antibody-dependent cellular phagocytosis^6,7^. Trastuzumab treatment has been shown to have several effects on the immune system, including increased natural killer (NK) cell proliferation and cytotoxicity, NK cell migration and adhesion, and increased phagocytosis of trastuzumab coated tumour cells by tumour-associated macrophages (TAMs), leading to increased tumour cell death^7,8^.

Tumour infiltrating lymphocytes have been associated with trastuzumab efficiency and improved survival in several cancers including breast cancer^9–11^. In our previous work with metastatic HER2+ breast cancer^12^, a high number of cytotoxic CD8^+^ T-cells in the center of the tumour, but not in the invasive margin, was significantly associated with the improved survival and patient’s ability to remain disease progression-free without trastuzumab after initially responding to the therapy.

Macrophages are known for their immunomodulatory effects. In a conventional manner, macrophages can be divided according to their phenotypes into M1- or M2-like states; M1-like macrophages have been linked to pro-inflammatory response, while M2-like macrophages are traditionally associated with wound repair and suppression of inflammation^13^. In many solid cancers, the number of TAMs is associated with prognosis and therapy response. In particular, TAMs with M1-like phenotype have been linked to good disease course while M2-like phenotype has been associated with adverse outcome, potentially through immunosuppression and the promotion of angiogenesis and tumour cell proliferation and invasion, which are critical prerequisite for metastatic cancer progression^14^.

In addition to M2-like macrophages, other cells of the immune system, such as regulatory T-cells (Tregs), can bare immunosuppressive functions. Tregs are essential for the development and maintenance of self-tolerance, preventing detrimental autoimmunity. On the other hand, Tregs have also been demonstrated to elicit protumour activity by suppressing antitumoural immune response. Tregs are in a reciprocal crosstalk with responding T-cells, M1/M2 macrophages and dendritic cells^15^. This crosstalk is mediated via contact-dependent cell-to-cell signalling and humoral signalling. Tregs are shown to utilize a variety of molecules to achieve immunosuppression, such as cytotoxic T-lymphocyte-associated protein 4 (CTLA-4), IL-2 and IL-10^16^.

Expressed by a subset of tumours and immune cells, negative immune regulatory molecules may contribute to the suppression of anti-tumour immunity. Indoleamine 2,3-dioxygenase 1 (IDO1, also called IDO) is an intracellular enzyme catalysing the degradation of tryptophan, which is essential for immune tolerance^17^. IDO1 is expressed by a population of antigen presenting cells such as some dendritic cells and macrophages. Furthermore, many human tumours have been shown to express IDO1, enhancing the suppression of effector T-cells and NK cells. Conversely, IDO1 promotes the formation and activity of Tregs^18–24^. Another intriguing signalling molecule is CD47, which is expressed by variety of malignancies and negatively impacts phagocytosis^25–27^.

In this current study, we evaluated immunological markers in pre-treatment tumour samples of metastatic HER2+ breast cancer patients receiving trastuzumab therapy. Our primary hypothesis testing was set to assess the association between different macrophage subtypes, the density of FoxP3^+^ Tregs, and survival, while all other analyses were secondary. The results suggest that M1 polarized macrophages together with cytotoxic T-cells found in the center of the tumour are strongly positive and independent indicators for prognosis and predict long progression-free periods without trastuzumab.

## Results

### Patients and samples

We identified 54 patients who had received at least one doze of trastuzumab for metastatic breast cancer in 2009–2014 from the Oulu University Hospital pharmacy records. Of these patients, 41% had primary metastatic disease at the time of diagnosis and 59% had a relapsed disease. Analyses were limited to pre-treatment samples, which were available for 40 patients. The samples included 25 (62.5%) surgical specimens and 15 (37.5%) biopsy specimens. Furthermore, 12 patients of the 40 (30%) had a planned HER2 therapy interruption in tumour response.

**Figure 1:**
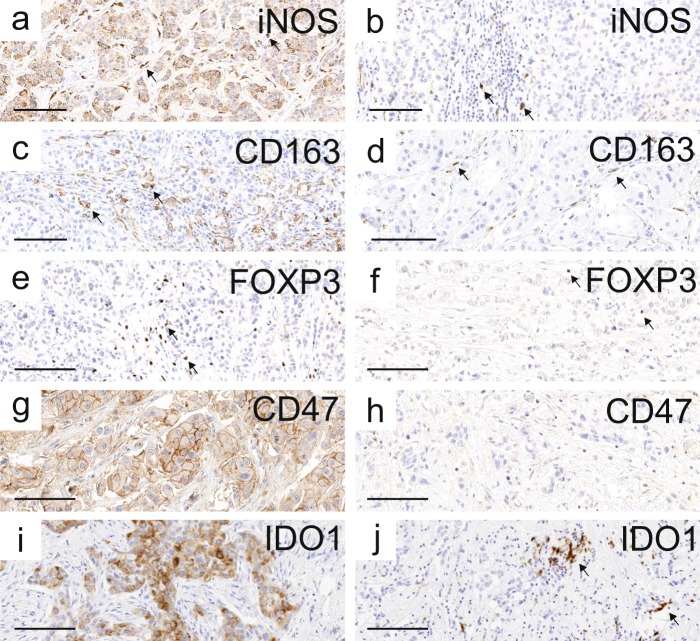
Representative examples of iNOS, CD163, FoxP3, CD47, and IDO1 immunohistochemistry. (**a**,**b**) iNOS positive and iNOS negative staining of the tumour cells. A subset of immune cells with macrophage-like morphology showed strong positivity. (**c**,**d**) High and low CD163 staining. A subset of immune cells with macrophage-like morphology showed strong cytoplasmic positivity. (**e**,**f**) High and low FoxP3 staining. Some lymphocytes had strong nuclear positivity. (**g**,**h**) CD47 positive and negative staining of the tumour cells. (**I**,**j**) IDO1 positive and negative staining of the tumour cells. In (**j**) but not in (**i**), some immune cells with macrophage-like morphology show strong immunoreaction. In all images, examples of positive immune cells are indicated with arrows. Scale bar = 100 µm.

The tumour samples were stained for inducible nitric oxide synthase (iNOS, M1 marker), CD163 (M2 marker), FoxP3 (Treg marker), CD47 and IDO1. Positivity for iNOS, CD163, and IDO1 was detected in a subset of tumour-associated macrophages (TAMs), while iNOS and IDO1 positivity was also seen in a subset (83% and 20%, respectively) of tumour cells (Fig. 1). FoxP3 positivity localized in the nuclei of a subset of lymphocytes, and membranous CD47 positivity was seen in tumour cells of 78% of tested cases (Fig. 1). Immune cells were analysed from the invasive margin (IM) and from the center of the tumour (CT) with a computer-assisted counting method previously used in colorectal cancers and HER2+ breast cancers^12,28^. CD47 and IDO1 staining in tumours was ranked from low (0) to high (3) intensities.

### Polarized macrophages predict survival

**Figure 2:**
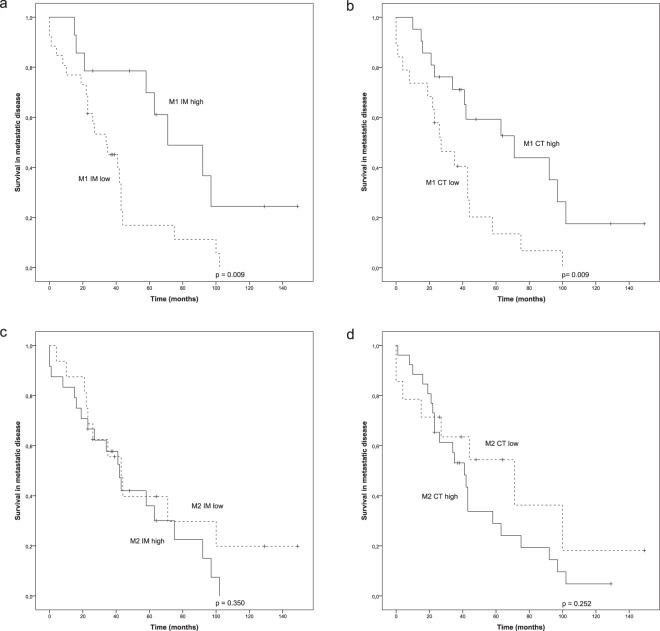
Survival analysis of metastatic HER2+ breast cancer in the presence of low or high infiltration of M1 and M2 polarized macrophages. (**a**,**b**) Kaplan-Meier estimates illustrate the association of M1 polarized macrophages with survival in IM (**a**) and CT (**b**) locations (cut-offs 42 and 37 cells/mm^2^). (**c**,**d**) Kaplan-Meier estimates illustrate the association of M2 polarized macrophages with survival in IM (**c**) and CT (**d**) locations (cut-offs 338 and 295 cells/mm^2^). Crosses mark censored events.

Differentially polarized TAMs were studied with iNOS and CD163, with a prespecified hypothesis that iNOS^+^ TAMs (M1-like phenotype) would be associated with improved prognosis, whereas CD163^+^ TAMs (M2-like phenotype) would be associated with adverse prognosis. iNOS^+^ and CD163^+^ cells were present in varying numbers in both IM and CT in all the tumours (Table 1). Kaplan-Meier estimates showed that a high number of iNOS^+^ M1-like macrophages in the IM (p = 0.009) and in the CT (p = 0.009) was significantly associated with an improved survival (Fig. 2a,b). In addition to M1-like macrophages, iNOS expression was detected in some tumour cells, but this did not provide any prognostic value (data not shown). The density of M2 polarized macrophages in the CT but not in the IM displayed a tendency towards poor survival, however, without statistical significance (Fig. 2c,d).

**Table 1:** Densities and receiver operating characteristics (ROC) analysis cut-off values of the studied immune cells.

		Median density (cells/mm^2^)	Range (cells/mm^2^)	ROC cut-off value (cells/mm^2^)
M1-like macrophages (iNOS)	IM	42	4–145	58
	CT	37	4–173	37
M2-like macrophages (CD163)	IM	338	24–957	307
	CT	295	15–983	241
Regulatory T-cells (FoxP3)	IM	145	14–739	134
	CT	92	6–796	82

We also studied whether a combined M1 and M2 status could predict the patient outcome even better than the high number of M1-like macrophages alone. High number of M1-like macrophages together with low number of M2-like macrophages in both CT and IM was associated with improved survival (CT: p = 0.032, IM: p = 0.045), however, these associations were not more significant than the high number of M1-like macrophages alone.

### Tumour immunosuppressive markers predict worsened survival

Next, we studied cells and molecules contributing to the immunosuppression in tumour microenvironment and their impact on survival. We hypothesized that high density of tumour infiltrating Tregs, and IDO1^+^ TAMs, as well as high tumour CD47 and IDO1 expression would be associated with adverse outcome.

**Figure 3:**
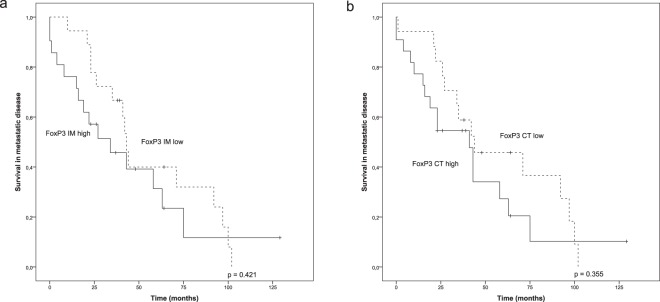
Survival analysis of metastatic HER2+ breast cancer in the presence of low or high infiltration of regulatory T-cells. Kaplan-Meier estimates demonstrate the association of regulatory T-cells with survival in the IM (**a**) and CT (**b**) locations (cut-offs 145 and 92 cells/mm^2^). Crosses mark censored events.

We found that variable numbers of Tregs, identified with FoxP3 nuclear positivity, were present both in the IM and CT in each tumour (Table 1). In the survival analysis, a high number of Tregs both in the IM and CT showed a tendency towards worsened survival (Fig. 3a,b), however not reaching a statistical significance.

**Figure 4:**
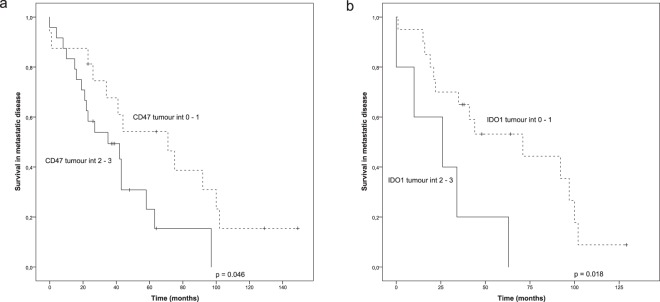
Survival analysis of metastatic HER2+ breast cancer in the presence of low (int 0–1) or high (int 2–3) tumoural expression of CD47 and IDO1. Kaplan-Meier estimates illustrate the association of CD47 (**a**) and IDO1 (**b**) intensities (int) in the tumours with survival. Crosses mark censored events.

Next, we evaluated potential immunosuppressive factors expressed by the tumour cells. Low CD47 intensity (0–1) in the tumour was associated with improved survival, whereas high CD47 intensity (2–3) predicted worsened survival (p = 0.046, Fig. 4a). Similar to CD47, tumour intensity of IDO1, a broadly studied immunosuppressive molecule, was used to group the samples into two categories (0–1, 2–3). IDO1 analysis was limited only to 25 tumours because of limited tumour sample availability. The results showed that high tumour IDO1 intensity was associated with poor survival (p = 0.018, Fig. 4b). IDO1 expression was observed also in a subset of macrophages, both in the IM and CT, but their density showed no association with survival (not shown). We also performed a combined analysis of the CD47 and IDO1 tumour cell intensities using three-tiered classification; high intensity of both markers, high intensity of only one marker, low intensity of both markers. A significant association was seen between the low intensity of both CD47 and IDO1 with improved survival (p = 0.037). However, in a multivariate analysis only IDO1 remained as an independent prognostic factor (CD47: HR 1.973, 95% CI 0.725–5.369; p = 0.183, IDO1: HR 3.333, 95% CI 1.108–10.031; p = 0.032).

### High number of tumour CD8 cells and M1 macrophages independently predict survival and long trastuzumab-free periods

**Figure 5:**
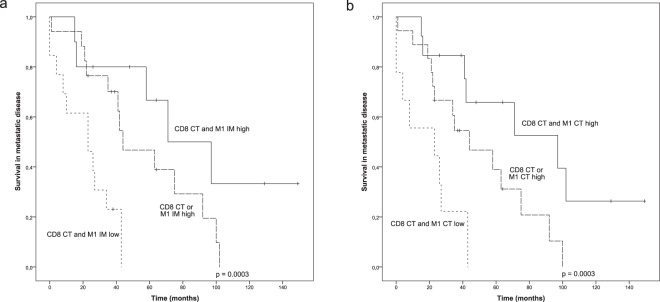
Survival analysis of metastatic HER2+ breast cancer in the presence of low, intermediate or high infiltration of both M1 polarized macrophages and CD8^+^ T-cells. (**a**) Kaplan-Meier estimate illustrates the association between M1 macrophages in the IM combined with CD8^+^ T-cells in the CT and survival. (**b**) Kaplan-Meier estimate illustrates the association between M1 macrophages in the CT combined with CD8^+^ T-cells in the CT and survival. Crosses mark censored events.

We have previously shown that a high number of CD8^+^ cytotoxic T-cells in the center of the tumour predicts better survival in metastatic disease^12^. Since in the current study, the quantity of iNOS^+^ M1-like macrophages was the most promising indicator for improved survival, we wanted to assess if combining these two markers would enhance the prognostic value compared to single markers alone. A high number of CD8^+^ T-cell in the CT combined with high number of iNOS^+^ M1-like macrophages in the IM (p = 0.0003, Fig. 5a) or CT (p = 0.0003, Fig. 5b) was significantly associated with improved survival compared to positivity by single marker. On the contrary, patients with both low CD8^+^ T-cells in the CT and low iNOS^+^ M1-like macrophages in the IM or CT had worsened survival compared to patients with single positive tumours (Fig. 5).

We further analysed whether CD8^+^ T-cells in the CT and iNOS^+^ M1-like macrophages in the IM or CT would remain as independent prognostic factors in a multivariate analysis. When the density of CD8^+^ T-cells in the CT (HR 2.923, 95% CI 1.305–6.546; p = 0.009) and M1-like macrophages in the IM (HR 3.078, 95% CI 1.298–7.298; p = 0.011) were included in the same model, both parameters remained as independent prognostic factors. Similarly, when the density of CD8^+^ T-cells in the CT (HR 2.957, 95% CI 1.336–6.543; p = 0.007) and M1-like macrophages in the CT (HR 2.813, 95% CI 1.302–6.077; p = 0.009) were included in the same model both remained as independent prognostic factors.

**Figure 6:**
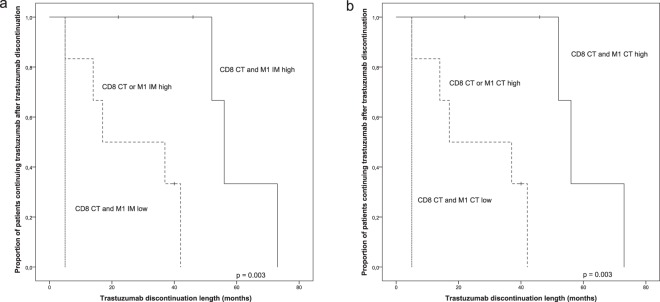
M1 polarized macrophages and CD8^+^ T-cells predict the length of trastuzumab-free period after response. (**a**) Kaplan-Meier estimate demonstrates the association of combined M1 IM and CD8 CT status with the trastuzumab discontinuation length. (**b**) Kaplan-Meier estimate demonstrates the association of the combined M1 CT and CD8 CT status with the trastuzumab discontinuation length. Crosses mark censored events.

Our previous results showed that a high number of CD8^+^ T-cells in the center of the tumour predicts patient’s ability to remain disease progression-free without trastuzumab after responding to the therapy^12^. We wanted to study whether the density of iNOS^+^ M1-like macrophages in the IM or CT combined with CD8^+^ T-cells in the CT would predict the length of trastuzumab free-periods in metastatic disease better than CD8^+^ T-cells alone. A high number of CD8^+^ T-cells in the CT together with a high number of M1-like macrophages in the IM (p = 0.003) or CT (p = 0.003) was strongly associated with a long trastuzumab free-periods after response (Fig. 6a,b).

## Discussion

The development of HER2 targeting antibodies, such as trastuzumab, have revolutionized the treatment and outcomes of *HER2* positive breast cancer patients^3–5^. However, trastuzumab is still administered according to the *HER2* status only due to a lack of predictive factors for trastuzumab sensitivity and resistance. Since the mechanisms of action of trastuzumab is related to both signal inhibition and immune activation^29^, trastuzumab responses can be affected by the altered signalling pathways and the immunological status of the tumour. Some mechanisms for the trastuzumab resistance have been described, such as compromised host immunoactivation, a shorter variant of HER2 (p95), and altered expressions/mutations of *PI3KCA* and *PTEN*^30^. We have previously shown a strong association between high number of CD8^+^ T-cells in the center of the tumour and improved survival and long trastuzumab-free periods in metastatic HER2+ breast cancer^12^. In the current study we investigated additional immunological markers which could be used together with CD8 for predicting the prognosis and trastuzumab responses in metastatic HER2 positive breast cancer.

Macrophages have been widely studied in different cancer types and their presence in the tumour microenvironment is known to have clinical relevance^31^. Current research of cancer immunology is leaning towards defining the role of macrophage polarization and its impact on the disease course and outcome. M2 polarized TAMs are known to have immunosuppressive functions and, indeed, M2 polarized macrophages have been correlated to poor prognosis in various solid cancers including breast, ovarian, gastric and colorectal cancers^32–35^. Conversely, antitumour M1 polarized macrophages are linked to favourable prognosis in lung, ovarian, gastric and hepatocellular cancers^34,36–38^. Little is known about the clinical importance of macrophages in HER2+ breast cancer, especially about M1 polarized macrophages and their prognostic role. We found that high density of iNOS^+^ M1-like macrophages predicted improved survival in our cohort of HER2+ breast cancer, which is consistent with other studies in other types of cancer. Our data highlights the potential of M1 polarized macrophages as a prognostic marker in metastatic HER2+ breast cancer. We have previously shown that prognostic role of CD8^+^ T-cells is limited to the center tumour location only^12^ while for TAMs, the number but not the spatial distribution, is significant. This might reflect the role of macrophages as a general regulator of tumour immunology. Our results indicate that therapeutic approaches targeting macrophage polarization in HER2+ breast cancer could represent a reasonable target for further investigation.

The function of trastuzumab is mainly mediated by immunoactivation^29,39^. Mechanistically, immunological function of tumour directed antibodies is thought to be principally caused by NK cell-mediated antibody-dependent cellular cytotoxicity. Previous studies with *HER2* positive breast cancer have demonstrated increased NK cell infiltration and activation in tumours after administration of trastuzumab and its relation to good responses^39–42^. A very recent work^43^ has highlighted the effects of macrophages on trastuzumab responses. Trastuzumab enhances antibody-dependent cellular phagocytosis by macrophages leading to upregulation of IDO1 and PD-L1 and immunosuppression. This finding is counterintuitive considering to the well-established benefit of monoclonal antibodies in cancer therapy and raises a question whether macrophages could be used as a predictive marker and therapeutic target. Our results favour the importance of macrophages for tumour immunology and their therapeutic exploration in HER2+ cancers.

Upregulation of IDO1 has been linked to high number of regulatory T-cells in tumours and increased M2/M1 ratio^22,44,45^ while high CD47 has been linked to inhibition of macrophage-mediated phagocytosis^25,27^ both leading to tumour immunosuppression. Our results also showed that low IDO1 and CD47 in tumour cells associated with improved prognosis, further supporting the importance of TAMs. A recent study^46^ has shown that blocking CD47 in addition to CD20 can have substantial clinical effect in lymphomas with very favourable safety profile. It is still early to conclude whether macrophage-mediated phagocytosis has strong immunoactivating or immunosuppressive function in tumours.

Trastuzumab still remains the gold standard for the treatment of HER2 positive breast cancer but acquired resistance develops in most patients with metastatic disease and some are primary refractory for the treatment. Currently, the use of trastuzumab in cancer therapy is determined by HER2 status only. In the era of personalized medicine, patient selection for the treatment should be better optimized. The results by us and others^47^ have clearly defined that immune cells have crucial impact on the disease course, and characterizing the amount and function of these cells could provide a new aspect for treatment selection. The patients with favourable tumour immunoprofile (high tumour infiltrating CD8^+^ T-cells and iNOS^+^ M1 TAMs in the CT, and low tumour expression of CD47 and IDO1) might be treated with less intensive manner and remain progression-free during trastuzumab interruption while patients with non-favourable immunoprofile are candidates for experimental immunotherapeutic approaches. However, these hypotheses need to be confirmed in subsequent larger prospective studies.

Our study had some limitations. The number of tumour samples was low, and patients were retrospectively collected from a single academic cancer center. Furthermore, tumour material of the study consisted of patients, all of whom were treated with trastuzumab, making evaluation of predictive values more difficult. Identification of immune cell phenotypes was carried out with single markers only, which does not acknowledge for the complexity of the phenotypically diverse group of macrophages^48^. However, well characterized and widely used markers were selected for the study. Multiple hypotheses were tested in this observational study, which increases the risk of type 1 statistical error. Therefore, we interpreted the findings with p near 0.05 with caution, and the significance of the validation of these findings in an independent study with larger patient material needs to be emphasized.

In conclusion, our study investigated the role of immunological markers in metastatic HER2+ breast cancer patients receiving trastuzumab therapy. The main results suggest that iNOS^+^ M1- like macrophages together with cytotoxic T-cells are independent indicators for prognosis and predict long progression-free periods without trastuzumab.

## Materials and Methods

### Patient data

The patients were retrospectively identified from the pharmacy records of Oulu University Hospital as previously^12^. All patients who had received at least one dose of intravenous trastuzumab in 2009–2014 for the treatment of metastatic breast cancer were selected (n = 54). The study was limited to those patients who had adequate pre-treatment samples available (n = 40). HER2 positivity was characterized by the presence of *HER2* amplification in chromogenic *in situ* hybridization. The patient data collection and tumour analysis were carried out under permits from the medical director of Oulu University Hospital (study no. 60/2015), the Northern Osthrobothnia Hospital District ethical committee (114/2011, amendment 23.02.2015) and National Supervisory Authority for Welfare and Health (9850/05.01.00.06/2010). According to the national legislation of Finland, tissues gathered for diagnostic purposes can be used in scientific studies without an informed consent from the patient and therefore it was not obtained, and it is not relevant for the study. All the experiments were performed in accordance with relevant guidelines and regulations.

The patients’ age, date of diagnosis, date of metastatic disease, adjuvant/metastatic treatment regimens, treatment durations and therapy responses among other characteristics described earlier^12,49^ were collected from the electronic patient records. Survival in metastatic disease was calculated from the time of histological or radiological identification of metastatic disease to death or end of follow-up. Patients whose HER2 therapy was interrupted were characterized by having a planned HER2 therapy interruption in connection with a response lasting >12 months or with a response plus severe suspected HER2 therapy-related adverse events. HER2 therapy discontinuation length was defined from the date of the last administration of trastuzumab (in the longest HER2 therapy discontinuation period) to the date of drug re-initiation, death, or end of follow-up. The clinical characteristics of the patients have earlier been described in more detail^49^.

### Immunohistochemistry and immune cell counting

Immunohistochemistry was conducted on 3.5 µm sections cut from paraffin-embedded specimens. The sections were deparaffinized in HistoClear (IDO1) or xylene and rehydrated through graded alcohols. Antigen retrieval was conducted in a microwave oven with citrate buffer (pH6; IDO1) or Tris-EDTA buffer (pH9; iNOS, CD163, FoxP3, CD47) at 800 W for 2 min and at 150 W for 10 (IDO1) or 15 min (other markers). Endogenous peroxidase activity was neutralized in 3% H_2_0_2_-aqua solution or in Dako REAL peroxidase blocking solution for 5 min. The tissue samples were incubated with primary antibodies (IDO1, Cell Signaling Technology, rabbit monoclonal, clone D5J4E™, dilution 1:400, overnight at + 4 °C; iNOS, Enzo Life Sciences, rabbit polyclonal, ADI-905–431–1, dilution 1:200, 60 min at RT; CD163, NeoMarkers, mouse monoclonal, clone 10D6, dilution 1:200, 30 min at RT; FoxP3, Abcam, mouse monoclonal, clone 236 A/E7, dilution 1:100, 30 min at RT; CD47, Atlas Antibodies, rabbit polyclonal, HPA044659, dilution 1:150, 60 min at RT). Bound antibodies were detected using the EnVision^TM^ (Dako) system. Diaminobenzidine (DAB) was used as the chromogen and hematoxylin as the counterstain. CD8 (Novocastra, mouse monoclonal, clone 4B11, dilution 1:200) staining used in the current study was performed earlier^12^.

For the analysis of immunohistochemistry, the sections were scanned with Aperio AT2 image-capturing device (Leica Biosystems). Imagescope (Aperio Technologies) software, version 11.2 was used to view the scanned images and capture images from the center of the tumour (CT) and the invasive margin (IM) for immune cell counting. The criteria for the different tumour locations are previously described^12^. The intensity of tumour cell immunoreaction for iNOS (cytoplasmic), IDO1 (cytoplasmic), or CD47 (membranous) was evaluated as negative (0), weak (1), moderate (2), or strong (3)^50^. The immune cells were counted using an earlier described and validated computer-assisted counting method^28,51^ that utilizes ImageJ, a freeware image analysis software^52^. Final counts presented in this manuscript are the average cell densities for each tumour area.

### Statistics

IBM SPSS Statistics 24.0 for Windows (IBM Corporation, Armonk, NY, USA) was applied for statistical analysis. The reported p-values are from two-sided chi-square tests. Receiver operating characteristics (ROC) analysis for the whole patient material was used to determine optimal cut-off scores of iNOS, CD163, FoxP3 and IDO1 for discriminating the survivors from the non-survivors. In the survival analyses, scores over the cut-off value were defined as “high” and the scores below were defined as “low”. Survival was analysed by using the Kaplan-Meier method with the log-rank test. Multivariate analysis was performed using Cox regression analysis. Probability values below 0.05 were considered significant.

## Data Availability

The raw data used in the analyses of the current study is not publicly available due to legislative issues concerning the privacy of the patients.
